# Digital redesign of the radiotherapy course for medical students with a blended learning approach

**DOI:** 10.1007/s00066-024-02348-x

**Published:** 2025-01-17

**Authors:** Anne Caroline Knöchelmann, Jan-Niklas Becker, Gerald Stiller, Diana Steinmann, Marianne Behrends

**Affiliations:** 1 Department of Radiotherapy, Hannover Medical School, Carl-Neuberg-Str. 1, 30625 Hannover, Germany; 2 Peter L. Reichertz Institute for Medical Informatics of TU Braunschweig and Hannover Medical School, Carl-Neuberg-Str. 1, 30625 Hannover, Germany

**Keywords:** E-learning, Medical education, Teaching evaluation, Radiation oncology, Teaching format

## Abstract

**Purpose:**

Due to the need for high-quality teaching, we present a new blended learning concept combining digital modules, interactive seminars, and clinical experience. Furthermore, we evaluated its acceptance among students.

**Methods:**

A new concept for teaching was applied to the radiotherapy module and made available via the Hannover Medical School (MHH) learning management system as part of a blended learning concept with educational films, multimedia learning modules, online seminars, and onsite practical training. The evaluation recorded assessments of the requirement profile, design, and opportunities for skills acquisition; evaluation of the seminar and internship; and questions on the overall assessment.

**Results:**

The new concept was applied to four passes in the fourth academic year. A total of 152 students completed the radio-oncology learning module, which was evaluated by 46 students. Forty students considered the course duration to be appropriate for the material covered, and most students stated that their prior knowledge had been sufficient. The level of difficulty of the content was rated by the students as somewhat too high. The design of the digital course, the opportunity to acquire skills, the seminar course, and the practical course were mostly rated positively.

**Conclusion:**

Through digital redesign, a teaching concept can be implemented that combines self-determined learning, professional exchange with doctors, and clinical practice experience. The concept can be transferred to other areas because it combines theoretical knowledge transfer with synchronous teaching and clinical experience. The results of the evaluation show that the students rated the new concept well.

## Background

In the National Competence-Based Learning Objectives Catalog for Medicine (NKLM) [1], radiotherapy is part of the core curriculum of human medicine. Given the importance of radiotherapy for treating oncological diseases, it is appropriate that students learn the basic principles of radiotherapy [2]. Consequently, there is a great need for high-quality teaching and training in radiotherapy, both during medical school and in later specialist training [3]. Nevertheless, the curricular part of radiotherapy in medical studies is rather underrepresented in Germany, and teaching takes place mainly in the form of lectures or seminars [4, 5]. A survey showed that interdisciplinary cooperation plays a major role for the participants. The network of different specialist disciplines paves the way for good interdisciplinary cooperation and better communication between therapists with regard to the treatment of oncological patients [6]. If students are sensitized to radiotherapy aspects during their study program and are well trained, they will also have a better basis for communication and cooperation with other specializations in the context of multidisciplinary collaboration, for example in the interdisciplinary tumor conference.

However, to enable in-depth learning and promote the development of skills, relevant teaching content should not only be taught theoretically. Rather, it is necessary to find didactic solutions that provide students with patient-oriented insights into the subject. Medical students also desire teaching with practical and interactive methods [7]. Owing to experiences during the coronavirus pandemic, digital offers are also increasingly being used [8], and the combination of online learning phases with face-to-face teaching is considered particularly promising for medical training [9]. When designing teaching content, however, contact between radiotherapy physicians and students should also be facilitated to make use of the positive aspects of medical role models with regard to professional behavior and to strengthen interest in radiotherapy [10].

Against this backdrop and given the noticeable fatigue among students regarding participating in lecture courses, which assign them only a passive role, the radiotherapy learning offer at Hannover Medical School (MHH) was completely revised for the 2023–24 academic year. Up to this point, student teaching in radiotherapy included a one-hour seminar in presence including a tour through the department. The supplementary lectures took place on site and in presence. The practical part and the possibility of working on radiotherapy case studies were not taken into account here. When the radiotherapy teaching program was redesigned, five criteria were to be met that are considered central to the quality of teaching. The course offers should achieve the following:

1. enable individual, flexible, and competence-based teaching of the central, theoretical learning content using digital learning materials;
2. provide patient-oriented insights into the concrete course of a treatment pathway in radiotherapy;
3. contain interactive learning opportunities with reference to clinical aspects;
4. provide opportunities for communicative exchange between doctors and students to increase the visibility of the doctors involved as medical role models; and
5. provide the opportunity to gain insight into everyday clinical practice to gain personal experience.

This paper presents how the teaching of radiotherapy was implemented with respect to the criteria mentioned and how the students evaluated the new learning offer.

The aim of this article is to describe the implementation of a new blended learning concept and to analyze the acceptance of this new teaching and learning approach among students.

## Methods

An interdisciplinary team of experienced lecturers from the Department of Radiotherapy and Special Oncology and experts from the fields of didactics and digital teaching was formed to redesign the teaching. Student assistants were also involved in the implementation of the concept. On the basis of the ADDIE model [11], a concept for redesign was developed after the current teaching situation was analyzed, which formed the basis for developing the new courses. The ADDIE model describes a process for course development that is divided into five steps: analysis, design, development, implementation, and evaluation [12]. The different phases of the ADDIE model in the context of the redesign of the radiotherapy course are described below. Thereby, the first three phases are assigned to the methodological approach, while the implementation and evaluation are assigned to the results.

### Analysis of the current situation

The first phase of the redesign entailed an analysis of the current situation, which included the identification of critical points and areas for improvement. It was essential to determine the exact amount of time previously dedicated to teaching and the content that was taught to establish the framework for the redesign. This phase also involved setting the main objectives for the revision. For instance, one objective could be that the amount of teaching time should remain unchanged for students.

Radiotherapy is taught at the MHH as part of the HannibaL model study program in the fourth year of study as part of the Radiology—Imaging Procedures, Radiation Treatment, Radiation Protection module. In one academic year, radiotherapy classes are held four times. Until the reorganization, teaching consisted of five 45-minute lectures and 2 hours of practical training in the Department of Radiotherapy and Special Oncology. It became increasingly apparent that student participation in the lectures was declining and that there was a growing desire for active forms of learning. For the new teaching concept, the time for knowledge transfer was therefore to be reduced to 135 min, which would correspond to three 45-minute lectures, in order to create time for an interactive seminar that would enable a more active form of learning.

To reduce the number of lectures without compromising the quality of knowledge transfer, all teaching content was critically evaluated, redundancies were identified, and the central teaching content was selected. In addition to the biological and technical basics, the radiotherapeutic treatment of selected clinical diagnoses (bronchial carcinomas, gastrointestinal tumors, central nervous system [CNS] tumors, prostate carcinoma, lymphomas) and urgent radiation indications should be taught. The tumor entities were selected according to their incidence in Germany [13] and the importance of radiotherapy in a multimodal treatment setting. The treatment of other tumor entities (such as gynecological or head and neck tumors) is covered in other modules. Information on side effects and late effects was designated as optional and supplementary learning material, as this mode focuses on teaching treatment concepts.

### Design and concept of the redesign

On the basis of the findings of the analysis and the definition of the goals, in the design phase, a new concept was developed and discussed with all lecturers. To enable flexible learning, the concept envisaged the implementation of all exam-relevant teaching content as online lecture recordings or as a multimedia learning module. As a practical introduction to the module, an educational film was also designed to convey the treatment steps in radiotherapy. The fundamentally new 2‑hour seminar was designed as a digital offers with flexibly retrievable (online) parts and scheduled parts on site. Case studies were created in preparation for the seminar, and clinical situations were constructed, which were prepared in advance by the students. As part of the seminar in the form of a video conference, these case studies were then discussed. The concept of the practical course within the clinic in small groups of up to 15 students now includes more practical aspects, such as creating a radiation mask.

**Fig. 1:**
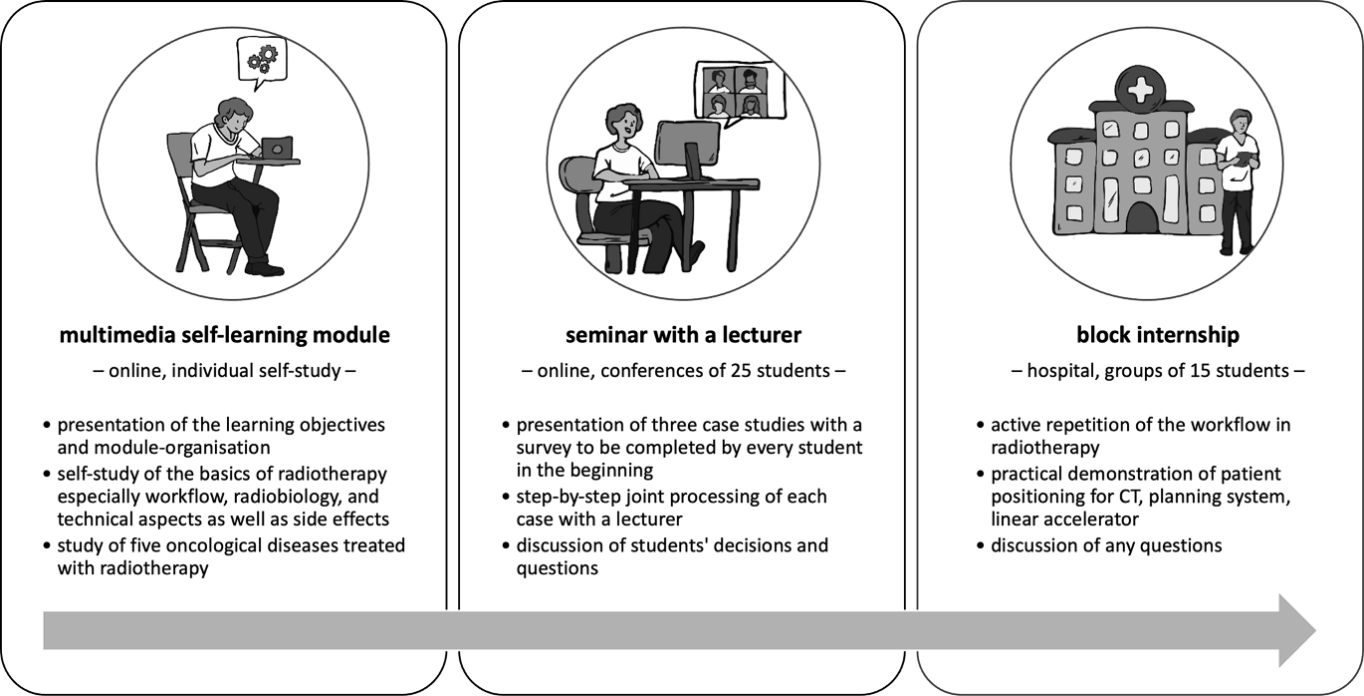
Components of the blended learning concept for the redesign of the radiotherapy course for medical students, enabling a different way of learning for acquiring the content and achieving the learning objectives

The concept for reorganizing teaching thus provided for a combination of different didactic elements: theoretical knowledge transfer through a multimedia self-learning module, opportunities for synchronous communication processes to apply the knowledge in the form of a seminar, and the acquisition of practical experience in everyday clinical practice through visits to the clinic (Fig. 1).

An example of the learning objective “Understanding the importance of radiation preparation and planning” can be found in the module as follows: Online, students are guided through the workflow and learn, for example, the indications for an irradiation mask and how it is made in practice, using text, graphics, and videos. This is then linked to corresponding clinical pictures, which are taken up again in the case studies of the seminars and discussed together. Finally, an irradiation mask is physically made with the students in the block internship in the appropriate sequence as part of the overall treatment process. The concept for the redesign, which envisaged an hour-neutral restructuring of curricular teaching for students, was submitted to the study commission and approved.

### Development of new learning content

In the development phase, all necessary learning materials were created, and the evaluation was designed. To create the learning materials, filming was carried out on the premises of the Department of Radiotherapy and Special Oncology in September and October 2023. To avoid disrupting the clinic’s routine, filming took place outside of the usual treatment times. Various scenes were filmed in different rooms of the clinic and then edited to create a chronological sequence of the radiotherapy treatment of a fictitious patient. Doctors and a Medical Technologist in Radiology (MTR) from the clinic played the protagonists in the film, with one doctor assuming the role of the patient. The off-screen commentary by a doctor explains a typical treatment procedure and the specific radiotherapy measures shown in the film. In the film, two doctors, as role models, guide the students through various stages of treatment: the film begins with the first sequence, in which the two doctors walk towards the camera in the reception area of the clinic and speak directly into the camera and, thereby, to the students. In further scenes, the doctors show and explain the individual steps of the treatment to the students, from the initial consultation, to planning of the radiotherapy and preparation of a radiotherapy mask, to radiotherapy of the patient. The presentation of the patient at an interdisciplinary tumor conference is also discussed. The students are given cinematic insight into various work processes and treatment rooms. The planning of computer tomography on the PC, the preparation of the radiation mask by the MTR for positioning the patient during radiation, the implementation of the therapy with the linear accelerator, and the debriefing with the patient are shown. The film ends with short statements by the doctors from the radiotherapy clinic. They formulate their individual views of everyday clinical work in radiotherapy and interaction with patients. The personal perspectives convey the special appeal of the subject.

Apart from this educational film, schemes for various treatment pathways have been created for clinical diagnosis and urgent radiation indications. The schemes are based on the clinic’s standard operation procedures (SOPs) for the respective clinical pictures, which are based on guidelines. Using these schemes, students can understand the respective radiation indications depending on tumor histology and spread diagnostics and independently develop treatment paths for patients. Thus, the role of radiotherapy can be illustrated particularly well in the context of multimodal treatment concepts. For each clinical picture, lectures of 10 to 15 min in length were recorded via schemes in which the doctors explained the most important steps and treatments in radiotherapy.

Short explanatory texts were written on typical side effects and late effects. Additionally, central care and treatment as well as integrative recommendations were made.

Furthermore, multimedia learning modules with texts, images, and videos were created for the biological and technical basics.

For the seminar, three case studies with typical clinical findings were prepared. All three cases describe critical situations, two of which are emergencies at the weekend. Questions concerning the suspected diagnosis, differential diagnosis, further procedures, and therapeutic options were developed for each case example. The case studies and associated questions were implemented as digital surveys in the MHH learning management system, so that the results of the survey can build the basis for discussion in the seminar. In the survey on the basis of various questions, the students are asked to make diagnostic and therapeutic decisions. The students do not receive immediate feedback on their decisions. Instead, all the student data are stored by the learning management system, automatically evaluated statically, and presented in diagrams. Teachers can view the results of the survey before the seminar begins and obtain a clear impression of the students’ assessments. In the subsequent online seminar, the students’ decisions regarding the cases are then presented by the lecturers, and the critical aspects are discussed with the students. Since all the students have already dealt with the cases in advance, their active participation in the seminar is supported.

All the digital parts were made available in the MHH learning management system. The entry page to the module was designed according to a scheme developed together with student assistants as part of the Lower Saxony joint project SOUVER@N—Sovereign Digital Teaching and Learning in Lower Saxony^1^.

Once the learning materials had been completed, a workshop was held before the start of the academic year with the four doctors conducting the seminars to introduce them to the didactic concept for conducting the seminars. The case studies were presented to the doctors in the workshop, and the seminar procedure was discussed with them. To ensure the uniform design of the seminars, the lecturers were asked to view the survey results concerning the three case studies before the synchronous case discussion began and to respond to the students’ selected answers in the seminar. The teachers also received a presentation with individual case studies.

### Evaluation concept

A questionnaire with 17 items related to various aspects was designed for the evaluation. The questionnaire recorded assessments of the requirement profile of the module, the design of the digital learning materials, and the possibility of acquiring skills through the learning materials. The evaluations of the seminar and the internship were also surveyed. There were also questions on the overall assessment and skills acquisition. It was also possible to make free-text comments. With respect to the students’ learning behavior, they were also asked whether they had worked on the digital learning content before the internship (Table 1).

**Table 1:** Overview of the items and the scale level of the questionnaire students were given to assess the new radiotherapy module

Item group	Item	Descriptions in the questionnaire	Answer categories
Requirement	Prior knowledge	My prior knowledge was sufficient to follow the radiotherapy module with the new digital courses	1 (fully applies)–6 (does not apply at all)
	Level	The technical level of the new digital teaching offers was for me…	1 (clearly too low)–6 (clearly too high)
	Time scope	The amount of time required to complete the digital learning was…	1 (reasonable); 2 (slightly too high); 3 (clearly too high)
Digital design of the module	Structure	The digital learning offer was clearly and unambiguously designed	1 (fully applies)–6 (does not apply at all)
	Motivation	The digital learning opportunities encouraged my interest in radiotherapy	
	Design	The digital learning offer was attractively designed	
Acquisition of technical skills through the learning materials	–	Thanks to the digital learning opportunities in radiotherapy, I was able to expand my skills in terms of…	–
	Basics	The principles and procedures of radiotherapy	1 (fully applies)–6 (does not apply at all)
	Fractionation schemes	The most common fractionation schemes	
	Technology	How an irradiation device works	
	Side effects	Side effects of radiotherapy	
	Indicators	Key indicators and common clinical pictures in radiotherapy	
Seminar	Deepening	The online seminar with the preparatory case processing helped to better understand the contents of the radiotherapy module	1 (fully applies)–6 (does not apply at all)
	Activity	I was able to contribute my knowledge in the online seminar with the preparatory case processing	
Internship	Deepening	The internship gave me an in-depth insight into the tasks and processes of radiotherapy	1 (fully applies)–6 (does not apply at all)
Overall rating	Increase in knowledge	The ratio of learning effort and increase in knowledge and skills through the digital learning offer was balanced	1 (fully applies)–6 (does not apply at all)
	Overall rating	Overall, I rate the design of the digital learning opportunities in radiotherapy with the school grade	1 (A)–6 (F)
Learning behavior	–	I worked on the learning content of the online module before the internship	1 (fully applies)–6 (does not apply at all)

The evaluation was carried out by the MHH evaluation office. At the end of each seminar, the lecturers informed the students of the opportunity to participate in the evaluation. The link to the survey was accessed via the learning platform. However, the evaluation results of the survey were recorded on a different platform. Teachers and those involved in the digital implementation did not have access to this platform. The evaluation of the modules was voluntary, anonymous, and not incentivized. After each implementation, the teaching staff and those involved in the digital implementation received the respective evaluation results. This should make it possible to react to any problems.

The feedback and usage figures for all four runs of the 2023/24 academic year were evaluated for this publication. Statistics and figures were generated using Microsoft Excel 2021 (Redmond, WA, USA). The analysis uses descriptive statistical methods to calculate the frequency distribution, median (mdn), and interquartile range (IQR) for all the closed questions. For the items with a six-point scale, the frequencies of the scales 1 to 3 were clustered as positive evaluations, and the frequencies of the scales 4 to 6 were summarized as critical attitudes. The answers to the open questions were summarized and evaluated by the last author. The access data from the learning management system were used to evaluate the usage figures.

## Results

### Implementation/realization in teaching

The newly designed module was run for the first time in the 2023/24 academic year. Therefore, the start page of the module in the learning management system provides students with an overview of the learning objective, information on the organizational structure of the module, contact persons, and the learning material. The learning material comprises the learning module, a seminar section with four groups per cohort, and some information on the internship at the Department of Radiotherapy. All course content relevant to the examination is summarized in a learning module.

The processing of all digital instructional content as a replacement for the lectures is estimated at three teaching hours of 45 min each. Completion of the survey of the three case studies and participation in the seminar are mandatory for students and are credited with 45 min each.

To conduct the seminar, each cohort was divided into four groups of up to 25 students. A separate group area was created in the learning management system for each group. In this group area, students can access the survey with the clinical decision cases. The link to the online seminar, which is the second part of the seminar, is also stored here. The seminars are held as synchronous online meetings, making it possible to involve external lecturers at other locations as teachers.

As part of a block internship, students were given the opportunity to familiarize themselves with the facilities and treatment steps in small groups of up to 15 people as interns. In particular, there was a practical demonstration of how to make a radiation mask, a planning CT with various positioning aids, a tour of the radiation machine linear accelerator (linac) with explanations, and a demonstration of the Monaco planning system (Elekta AB, Stockholm, Sweden) with the opportunity to contour in the planning system if interested.

Work shadowing in the radiotherapy clinic offered the students the opportunity to experience and deepen the content of the educational film and the theoretical learning content of the learning module through a learning spiral.

### Usage figures

**Fig. 2:**
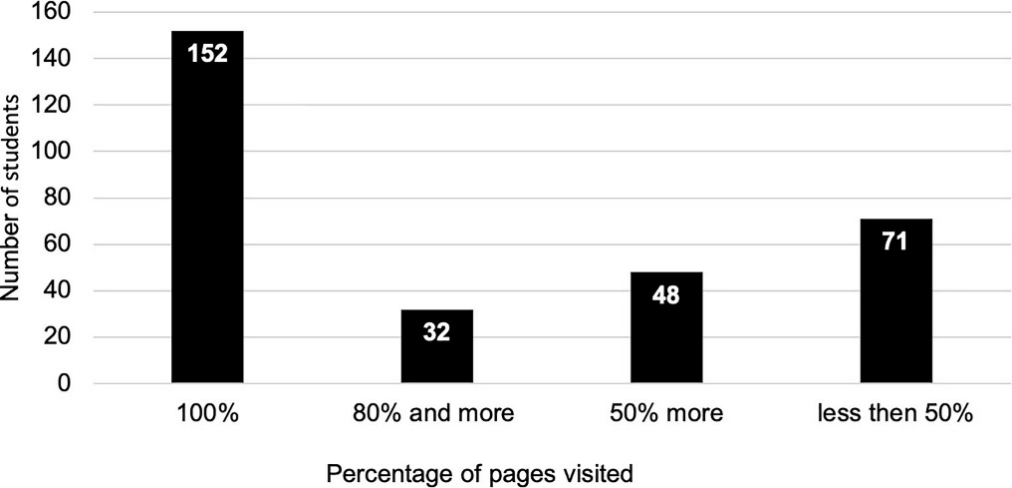
Frequencies of the usage of the digital learning content by the students based on the percentage of visited pages of the learning module according to the log files from the learning management system (*n* = 303)

**Fig. 3:**
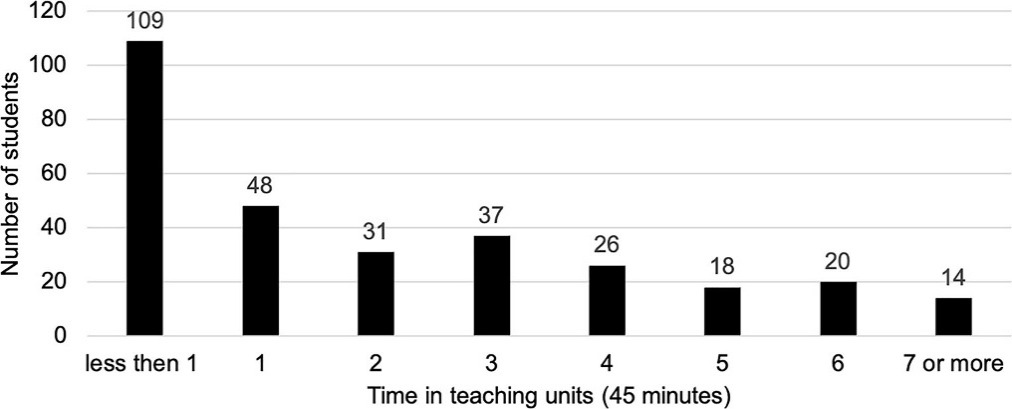
Frequencies of the usage of the digital learning module by the students in 45-minute intervals according to the log files from the learning management system (*n* = 303)

A total of 351 students in four cohorts were assigned to the lessons. According to the usage times recorded by the learning management system, 152 students visited all the pages of the learning module, and 32 students visited more than 80% of the pages (Fig. 2). The average completion time was 2:05 h, which corresponds to approximately three teaching units. Clustering according to intervals of lessons (45 min) shows a large variance in usage (Fig. 3). A total of 115 students spent three or more lessons on the learning module.

**Fig. 4:**
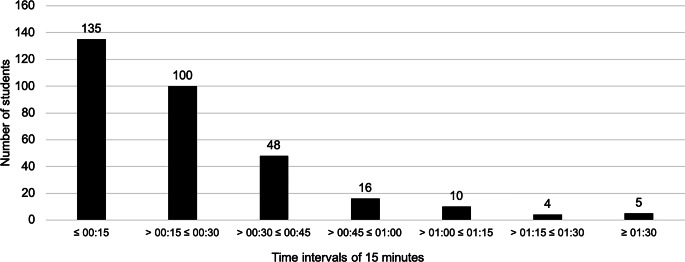
Frequencies of the processing times of the survey with case studies on clinical decisions (*n* = 318)

The usage figures show wide variation in the time taken to complete the survey as the first part of the seminar (Fig. 4). The clustering according to intervals of 15 min shows that 235 students spent less than 30 min working on the survey, while 19 students spent more than 1 hour. Sixty-four students spent between 30 and 60 min answering the questions.

With respect to the specified duration of the case processing survey, 283 of the participants worked on it for less than 45 min, while 35 students worked on it for longer, in some cases for more than 1:30 h.

### Evaluation results

A total of 46 students participated in the evaluation, and 43 evaluations could be used for the statistical analysis. Three students objected to their data being evaluated for publication. The response rate in relation to all students in the academic year was 13%. Thirty-two of the students surveyed were female, 10 were male, and one person did not specify their gender. Seventeen students entered free text.

Forty (93%) of the students considered the amount of time to be appropriate, and most students (35; 81%) stated that their prior knowledge had been sufficient to follow the module. The level of difficulty of the content was assessed by the students as slightly too high (mdn 4, IQR = 0).

All aspects related to the design of the digital offer, the opportunity to acquire specialist skills, the seminar, and the internship were rated positively by most students. The median score for all the questions was 2. The evaluation results are summarized in Table 2.

**Table 2:** Student evaluations

		Positive evaluation (scale values 1–3)	Critical attitude (scale values 4–6)	Positive rating %	Critical attitude%	Median	IQR	*n*
Design of the digital offering	Structure	39	4	90.7	9.3	2	1	43
	Motivation	37	6	86.0	14.0	2	1	43
	Design	37	5	88.1	11.9	2	1	42
Acquisition of technical skills through the learning materials	Basics	40	3	93.0	7.0	2	1	43
	Fractionation schemes	35	8	81.4	18.6	2	1	43
	Technology	38	5	88.4	11.6	2	1.5	43
	Side effects	41	2	95.3	4.7	2	1.0	43
	Indicators	39	3	92.9	7.1	2	1	42
Seminar	Seminar deepening	38	5	88.4	11.6	2	2	43
	Seminar activity	36	7	83.7	16.3	2	1	43
Internship	Internship deepening	38	5	88.4	11.6	2	1	43

The ratio of learning effort to increase in knowledge and skills through the digital learning offer was rated as balanced by 37 of the respondents (86%), with a median of 2 (standard deviation [SD] 1.1). The overall assessment score of the module by the students was 1.8 on average (SD 0.7). Most students (33; 76.7%) worked on digital learning materials before the internship.

The free-text comments contain observations on the learning materials, seminars, internships, lecturers, and organizations. Some central comments are given here with reference to the anonymized identity (ID) of the person interviewed. One student (ID38) noted that with respect to the time-independent availability of the teaching videos, the teaching material can now be repeated again before the exam, and teaching videos are therefore watched more often than face-to-face lectures. The “form with texts and instructional videos makes it easier to work through the subject matter” (ID9). The module is “well and clearly designed” (ID43). The seminars were mentioned in seven comments. The seminar was described as “very exciting” (ID6) and “very interesting and varied” (ID10), especially when all answer options were “discussed and very carefully and comprehensibly explained” (ID6). The evaluation of the seminars was decisively influenced by the evaluation of the lecturers. The lecturers were explicitly mentioned positively in four comments. However, there were also points of criticism regarding the implementation of the seminars: “Since the students have dealt with the topic and watched the videos beforehand, it would have been better to work out the individual answers with the students by asking questions” (ID3). Other points of criticism related to organizational aspects. For example, it was criticized that the timetable, which was created separately for the students in another program, contained incorrect time information or was missing. Two students also wished for a greater proportion of face-to-face teaching (ID9, ID24). Specific suggestions for improvement included making the learning material more interactive with small quizzes, key feature cases and assignment tasks (ID33), and completing each case individually when working on cases for the seminar (ID13).

## Discussion

The aim of the new concept for the radiotherapy course was to use digital learning material to make teaching flexible, interactive, and patient oriented for students. By introducing a seminar, it also sought to facilitate greater exchange with lecturers to offer insights into everyday clinical practice and increase the visibility of the doctors involved as medical role models. Due to the highly structured and fixed timetable for students, self-directed learning promotes flexibility and can be easily incorporated into everyday life by students. The ability to access the content flexibly in terms of time thus improves the integration of learning into everyday life. The practical application of the acquired specialist content demonstrates the subject of radiotherapy to the students in a clinical setting and raises awareness of the clinical relevance of the subject in everyday life.

The concept combines three different didactic approaches:

- the digital provision of core subject content enables self-determined and flexible learning;
- activating learning tasks in conjunction with seminars encourages reflection and consolidation of the learning material and provides opportunities for communication processes with the doctors; and
- practical work in the clinic enables personal exchange with doctors and makes everyday clinical practice tangible.

The three didactic approaches are not separate from each other but are interwoven in terms of content, methods, and personnel. For example, knowledge transfer begins with a film in which radiotherapy doctors present their clinical work, treatment, and workrooms, which are taken up again during the students’ visit to the clinic and are combined with real experiences. The theoretical content is taught by the same doctors, who accompany the seminars on clinical decision-making and practical training in the clinic, whereby the content also links theory and practice. By interweaving theory, critical exchange, and clinical experience, the doctors involved are able to convey the entire spectrum of radiotherapy to the students.

The evaluation of the new course by the students shows a consistently positive response. The majority of the students rated the design of the digital offer, the opportunity to acquire specialist skills through the learning materials, the seminar, and the practical course positively. Points of criticism primarily relate to organizational aspects and the lecturers’ performance. A structural anchoring of the digital course offers within the fixed timetables appears to be important for the students despite the flexibility of learning. Vorwerk et al. [14] also noted in their study that a clear temporal structure of the digital learning offer is important for students’ learning behavior.

Two students expressed the wish that teaching and seminars should take place in person again to facilitate better communication. On the other hand, various comments from students emphasized exchanges with lecturers in the online seminar. Some students also rated the fact that the learning materials were available at all times—even before the exam—as particularly positive. The fact that students’ attitudes towards digital teaching are not always uniform is also shown in studies by Dapper et al. [9]. They concluded that the future of medical teaching seems to lie in a combination of learning videos and practical seminars to enable flexibility and practical relevance. For Vorwerk et al. [14], digital teaching should ideally take place in combination with face-to-face teaching to give students the opportunity to ask questions. However, our results indicate that an online seminar in combination with a digital learning task to prepare for the seminar also enables a stimulating and learning-promoting exchange. How well this ultimately works depends on the teacher’s performance, as suggested by the results of this study.

Overall, the introduction of the seminar proved to be a target-oriented decision to promote active learning. This also corresponds to the findings of Wade et al. [15]. In their review, they demonstrated that didactic approaches for active learning yield better results in terms of knowledge acquisition and/or student engagement. The inclusion of interactivity, practical exercises, repetition, and feedback has been shown to promote learning outcomes. For higher semesters, the inclusion of “practice questions” seems useful. In addition, the case studies in the seminar present a kind of simulation of situations for clinical decision-making, which enables deliberate practice for the students. In their meta-analysis, McGaghie et al. [16] have already shown that in other disciplines, simulation-based medical education with deliberate practice can be more effective than traditional clinical education and should be used more widely. To the best of our knowledge, however, there are still few evaluation results available in this area, and new developments in teaching should be closely monitored and regularly adapted to the needs of students as part of evaluations.

The seminars, as well as the involvement of doctors in the educational film, also increased the visibility of doctors and, thus, the attractiveness of the subject of radiotherapy, as positive role models of doctors can have a positive effect on career decisions for a particular subject. This is shown by the results of the review by Lamb et al. [17], in which communicative skills and enthusiasm for teaching are key criteria for a positive role model.

The low usage figures for digital learning opportunities must certainly be viewed critically. Most students spent less time working on the learning materials than planned. However, as time measurement is very imprecise for technical reasons, especially when instructional videos are used in the learning management system, it must be assumed that the actual usage time is greater. Furthermore, not all the students accessed the learning materials. It is possible that students used the questions from the previous year to prepare for the exam and, therefore, did not use the digital learning material provided. Vorwerk et al. came to a similar conclusion when assessing the usage figures of their digital offers [14]. It is also possible that the students worked on the learning materials together, whereby only one person logged on to the computer, as the use of the learning materials was not mandatory. However, Oertel et al. reported that the digital conversion of teaching did not lead to lower student participation [8]. At the MHH, approximately 5–10 students attended radiotherapy lectures before the change in teaching. The new learning module was completed in full by 152 students, indicating an increase in engagement and suggesting a positive effect.

The concept of this blended approach can also be transferred to other areas, as it combines theoretical knowledge transfer with synchronous interactive teaching and experience in a real clinical setting on site. Particularly in subjects where a high level of specialist expertise is required and this specialist knowledge has to be transferred to practical work and decision-making processes, the linking of theoretical knowledge acquisition and clinical application is crucial. This includes clinical subjects, particularly in areas where theoretical knowledge has an impact on practical skills and manual skills are not primarily taught as part of the curriculum. However, due in particular to the fact that radiotherapy plays a rather subordinate role in the training of medical students, the creation of educational films and exercise units can also be used across locations, especially in the virtual area, and thus improve the training of students through cooperation projects in teaching. Other specialized disciplines are also already calling for new and innovative teaching formats to improve networking between the individual departments and also to adequately convey the breadth of medical content [18].

A criticism is that developing the new teaching concept required considerable effort. Mirestan et al. [19] noted, however, that a lack of knowledge of radiobiology, radiation physics, and radiotherapeutic treatment of oncological diseases in the curricula of medical faculties can have detrimental consequences for the training of residents in radiotherapy and for the choice of specialty after graduation. In this sense, efforts to improve teaching are also investments in the future of the discipline.

## Conclusion

The digital redesign of radiotherapy teaching at the MHH has made it possible to implement a teaching concept that combines self-determined learning, professional exchange with doctors as role models, and an experience of everyday clinical practice. The concept can also be transferred to other areas. The results of the evaluation also show that the students rated the new concept well.

## Footnotes

1 The project SOUVER@N—Digitales Lehren und Lernen in Niedersachsen (https://www.souveraenes-digitales-lehren-und-lernen.de/home/) is funded by the Stiftung Innovation der Hochschullehre (funding number: FBM2020-VA-143-8-01739).
